# Stress–Strain Model for Lightweight Aggregate Concrete Reinforced with Carbon–Polypropylene Hybrid Fibers

**DOI:** 10.3390/polym14091675

**Published:** 2022-04-20

**Authors:** Xue Yang, Tao Wu, Xi Liu

**Affiliations:** School of Civil Engineering, Chang’an University, Xi’an 710061, China; ms_yangxue@163.com (X.Y.); lliuxii@163.com (X.L.)

**Keywords:** compressive stress–strain relationship, lightweight aggregate concrete, carbon fiber, polypropylene fiber, hybrid fiber reinforcement

## Abstract

This research aimed to investigate the hybrid effects of carbon and polypropylene fibers on the stress–strain behavior of lightweight aggregate concrete (LWAC). The considered test variables were two fiber volume fractions of 0.2% and 0.4% and two water/binder ratios of 0.27 and 0.30. Eighteen groups of prisms fabricated with fiber-reinforced LWAC were tested under axial compressive load. Experimental studies were carried out to analyze the influence of different fiber combinations on the complete stress–strain behavior. It was found that the carbon–polypropylene hybrid fibers led to toughness enhancement that was numerically more significant than the sum of individual fibers, indicating a positive synergistic effect between them. Finally, a mathematical expression of the stress–strain curve accounting for the fiber combinations was developed. Compared with existing stress–strain models, the proposed model shows better accuracy in predicting the effect of carbon and polypropylene fibers in both single and hybrid forms on the stress–strain curve of LWAC.

## 1. Introduction

Lightweight aggregate concrete (LWAC) has been successfully used in structural engineering due to its advantages over conventional concrete, including lower density, superior thermal insulation, and higher specific strength [1,2]. However, disadvantages such as higher brittle texture and lower mechanical properties have restricted the wide range of applications for LWAC [3]. Incorporating fibers into concrete as a single or hybrid form is confirmed as an effective way of compensating for the adverse effects of LWAC. As is known, the most beneficial characteristic of fiber is the crack-bridging mechanism, which can significantly increase the toughness and post-cracking ductility of concrete [4,5,6].

Different types of fibers, such as steel, glass, carbon, nylon, and polypropylene, have been used to produce fiber-reinforced LWAC (FLWAC) [7]. Adding steel fiber to LWAC leads to a significant improvement in the mechanical performance of concrete but increases the density of LWAC. Instead, the incorporation of non-metallic fibers (carbon, basalt, polypropylene, etc.) in concrete has become attractive because of the high performance and low density [8,9]. LWAC comprising two or more types of fiber was investigated to achieve a positive synergistic response. Therefore, combinations of high-strength carbon fiber and high-ductility polypropylene fiber are hoped to reinforce LWAC at multiple scales.

The stress–strain relationship is crucial to the axial design of short columns and the flexural designing of slabs and beams [10,11]. Moreover, a thorough understanding of comprehensive compressive stress–strain behavior is essential for the nonlinear analysis of structures and the derivation of design behavior curves [10]. However, the effect of non-metallic fibers on the compressive stress–strain response of LWAC may be different from that on the properties of normal-weight concrete (NWC). To design and analyze the performance of the structural application of LWAC reinforced with carbon and polypropylene fibers, it is necessary to investigate relevant mechanical properties and propose a stress–strain model.

Continuing efforts regarding experimental and theoretical research have been dedicated to generating compressive stress–strain curves, and several empirical models have been established in the last few decades, such as those proposed by Carreira and Chu [12], Guo [13], and Wee et al. [14]. It was reported that the stress–strain response of concrete depended on the concrete composition [15]. Nevertheless, the constituents of LWAC differ from those of NWC, and it is uncertain whether a model proposed based on NWC can provide good predictions for LWAC. Meanwhile, the effect of fibers is not taken into account in the parameters of the proposed models.

Studies on the compressive behavior of fiber-reinforced NWC as well as LWAC have been carried out. Tasnimi [16] derived a stress–strain model applicable to both LWAC and NWC, but it did not reflect the properties of lightweight aggregate (LWA). Recently, a unified model was developed by Lim and Ozbakkaloglu [17] to describe the stress–strain behavior of both NWC and LWAC, but the proposed model is unsuitable for fiber-reinforced concrete. Wang et al. [18] proposed a model to predict the compressive stress–strain behavior of concrete reinforced with basalt–polypropylene hybrid fibers. Currently, the available evidence regarding the axial compressive behavior of FLWAC is still insufficient. The axial compressive behavior of LWAC reinforced with non-metallic fiber has yet to be investigated, and it is essential to develop a model for the prediction of this concrete.

This study explores the effects of single and hybrid carbon and polypropylene fibers on the compressive stress–strain behavior and model of LWAC. From the experimental results, analytical expressions for compressive strength and critical strain accounting for the fiber combinations were proposed. Finally, to capture the complete stress–strain response of FLWAC, a stress–strain model suggesting the peak stress and critical strain was derived.

## 2. Experimental Details

### 2.1. Materials

Lightweight Aggregate: Artificially expanded shale ceramic with crushed shape provided by Guangda Co., Ltd. (Yichang, China) was selected as coarse LWA. According to Chinese Specification GB/T 17431.2-2010 [19], its properties and particle size distribution are provided in Table 1. The chemical composition of LWA provided by the manufacturer is also listed in Table 1.

Fibers: Two types of fibers, namely, carbon and polypropylene fibers, were used, provided by Anjie composite material factory (Haining, China) and Hansen Co., Ltd. (Wuhan, China). Their respective features and properties, as provided by the suppliers, are listed in Table 2.

Binder: Ordinary Portland cement, classified as P.O 42.5, was used, complying with Chinese Specification GB 175-2007 [20]. Silica fume and class F fly ash in accordance with GB/T 18736-2017 [21] were added as active mineral admixtures.

The fine aggregate used was medium sand sieved to 4 mm with a bulk density of 1530 kg/m^3^.

A high-performance polycarboxylate-based superplasticizer was employed in all mixtures to improve fiber dispersion and adjust the fluidity in fresh LWAC mixtures.

### 2.2. Preparation and Details of Specimens

The effective water/binder ratio (W/B) employed was 0.27 and 0.3 for Series 1 and Series 2, respectively. Nine concrete mixtures were prepared with the same binder, aggregate, water, and superplasticizer for each series. In addition, silica fume and fly ash were applied to replace 0.08 and 0.12 by mass of cement, respectively. The mix proportions of plain LWAC are provided in Table 3. Accordingly, as shown in Table 4, different combinations of carbon and polypropylene fibers (volume fraction = 0.2% and 0.4%) were incorporated into the plain LWAC mixtures to investigate both the individual effects and synergistic effect of fiber reinforcement.

The concrete was prepared using a forced-action mixer under lab conditions. Before mixing, LWA was first pre-wetted with additional water to ensure an SSD condition. Firstly, powder-type ingredients such as cement, fly ash, silica fume, and medium sand were dry mixed for about 3 min. Next, polypropylene fibers were added and mixed in dry state for another 2 min, and after that, a half-quantity of pre-mixed water with superplasticizer was introduced to the dry mix. The mixture was mixed for a further 3 min, followed by the addition of pre-soaked LWA. To compensate for the absorbed water of carbon fiber, it is suggested to first blend carbon fiber with the remaining water to ensure sufficient dispersion. Subsequently, 3 min after the aggregate was added, the remaining water was gradually poured into the mixer. Mixing was performed for another 3~5 min until a consistent mixture was obtained. Simultaneously, fresh concrete mixtures were cast into standard molds. All specimens were compacted on a high-frequency vibrating table for 30 s and kept covered with plastic sheets for 24 h before demolding. The hardened specimens were cured in a standard curing room for 28 days and then placed at room temperature until testing time. For each concrete mixture, three 100 mm cubes were cast and cured under the same conditions as the specimens to determine the cubic compressive strength in accordance with the Chinese Specification GB/T 50081-2007 [22].

### 2.3. Experimental Instrumentation and Methods

**Figure 1:**
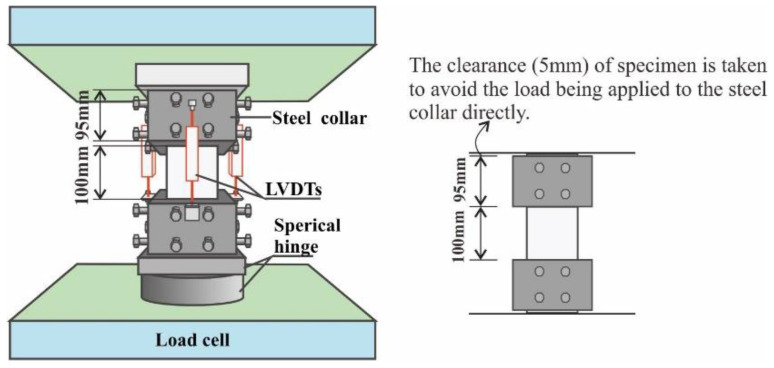
Scheme of axial compression test.

Axial compressive tests were performed on 100 mm × 100 mm × 300 mm prismatic specimens in compliance with the Chinese Specification GB/T 50081-2007 [22]. The specimens were tested on a 1000 kN electro-hydraulic servo universal testing machine at a displacement control rate of 0.02 mm/min. To avoid the end of concrete crushing, steel collars were employed to confine the top and bottom of the specimen. The axial deformation was measured using four linear variable displacement transducers (LVDTs) that were mounted at 90° around the test section of the specimen (see Figure 1). Before testing, the specimen was preloaded from 0 to 5 MPa and then unloaded to 0.5 MPa, which formed one loading–unloading cycle. Thus, more than three cycles were applied to the specimens to guarantee axial loading and prevent the slackness of the system [23]. During the test, the applied load and corresponding axial deformation were continuously recorded at a sampling frequency of 10 Hz to describe the stress–strain curve of the specimen.

Compressive stress was the applied load divided by the cross-sectional area of each specimen, and then compressive strain was calculated from the average value of four LVDTs. With the stress and corresponding strain, the experimental stress–strain curves can be plotted. Compressive strength (*f*_c_) and critical strain (*ε*_c_), corresponding to the peak point, were obtained directly from the experimental stress–strain curve. The initial tangential elastic modulus (*E*_c_) and peak secant elastic modulus (*E*_0_) were calculated using Equations (1) and (2), respectively:

(1) $$E_{c}=\frac{\sigma_{c2}-\sigma_{c1}}{\varepsilon_{2}-0.00005}$$

(2) $$E_{0}=\frac{\sigma_{c}}{\varepsilon_{c}}$$

where *σ*_c2_ is the compressive stress at the point of *f*_c_/3; *σ*_c1_ is the compressive stress at the point of the strain of 0.0005; and *ε*_2_ is the compressive strain at the point of the stress of *σ*_c2_.

## 3. Results and Discussion

### 3.1. Failure Mechanism

**Figure 2:**
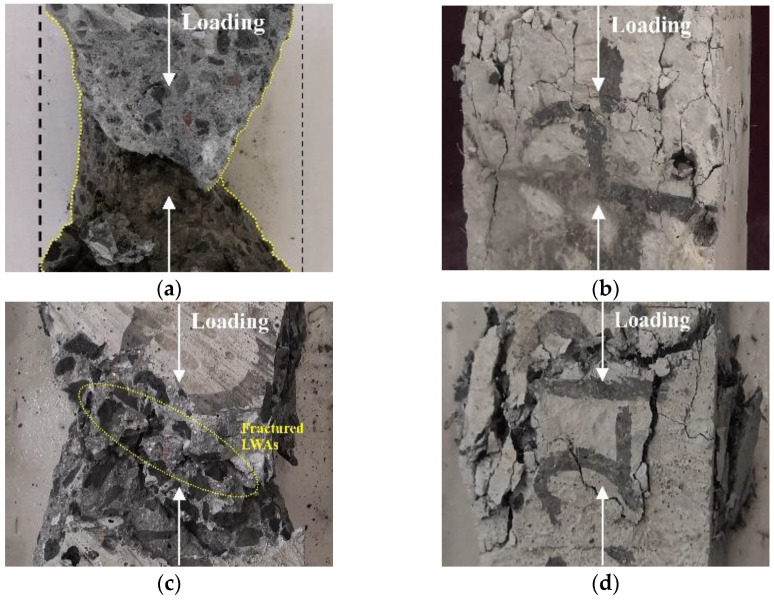
Failure modes of specimens under axial compression (W/B = 0.3): (**a**) Plain LWAC; (**b**) PFLWAC; (**c**) CFLWAC; (**d**) HFLWAC.

The effect of fiber reinforcement on LWAC can be directly characterized by the modification of the crack pattern and the failure mode. Specimens containing the same fiber combination but with different W/B show similar failure modes, such as Plain–a and Plain–b, so the failure mode of specimens with W/B of 0.3 was taken as an example to analyze the effect of fibers on the concrete failure mechanism. As shown in Figure 2, failure modes for carbon-fiber-reinforced LWAC (CFLWAC) and polypropylene-fiber-reinforced LWAC (PFLWAC) expressed significant differences from those of hybrid-fiber-reinforced LWAC (HFLWAC). In the specimen of plain LWAC, noticeable concrete crushing can be recognized from dense cracks forming after a compressive test. The failure mode of specimens of LWAC with only carbon fiber was similar to that of plain LWAC, whereas the degree of concrete spalling was relatively lighter for concrete containing carbon fiber. Cracks extended through the LWA were accompanied by the rupture of carbon fiber, which can be attributed to the low strength of LWA as well as the low ductility of carbon fiber. Moreover, fractured LWA on the crack surface can also explain the higher brittleness of LWAC as compared with NWC with the same strength grade.

In the case of LWAC containing polypropylene fiber, the specimen maintained integrity during the testing. However, there is a beneficial modification on the failure pattern in that there is no apparent concrete spalling, but dense cracks can be observed. This phenomenon may be due to the bridging effect of polypropylene fiber that restrains the cracking and lateral deformation of concrete. In addition, the ruptured polypropylene fiber can be viewed on the crack surface because of the high ductility of the polypropylene fiber, which allows a large deformation when subjected to tensile stress. Additionally, the difference in compressive strength was taken as partly responsible for the difference in failure modes between CFLWAC and PFLWAC. The compressive strength of LWAC containing carbon fiber was much higher than that solely containing polypropylene fiber.

### 3.2. Compressive Stress–Strain Behavior

**Figure 3:**
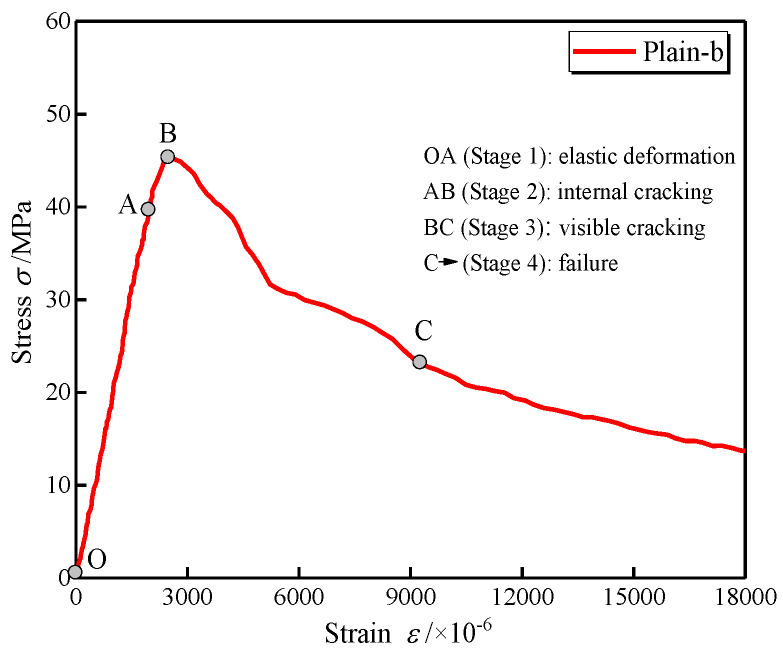
A typical example of the compressive stress–strain curve.

Figure 3 shows the typical stress–strain curve of LWAC in compression. During the loading procedure, the specimen’s response to axial compression was recorded to illustrate crack patterns corresponding to different stages in the stress–strain curve. As shown in the figure, four cracking stages during the compressive tests can be identified in the stress–strain curve. In stage 1, the stress can be assumed to be linearly increasing from point O to point A, illustrated by the elastic deformation of the aggregate and the cement paste. This linearity is maintained until approaching 90% of the peak point and is considerably larger than that of NWC (30~45%) [24]. Microcracks that existed before loading would not extend until the end of this stage. Point A is recognized as the point where the stress–strain curve deflects from linearity. Meanwhile, the cracks tend to propagate from point A. During stage 2, the strain increases more quickly than before, manifesting as a continuous decrease in the curve’s gradient. As the stress steadily increases, vertical cracks appear on the specimen until one major crack reaches its critical length at point B. Point B is referred to as the peak point, from which the peak stress and critical strain can be obtained. In the post-peak region, the specimen enters the cracking instability stage, and cracks propagate automatically, though the stress is decreasing. The duration of stage 3 is relatively short, as the stress decreases rapidly to about 50% of the peak stress, suggesting the brittleness of LWAC. Point C is referred to as the inflection point in the descending branch. In the case of stage 4, the load capacity mainly consists of the frictional resistance and residual stress.

**Figure 4:**
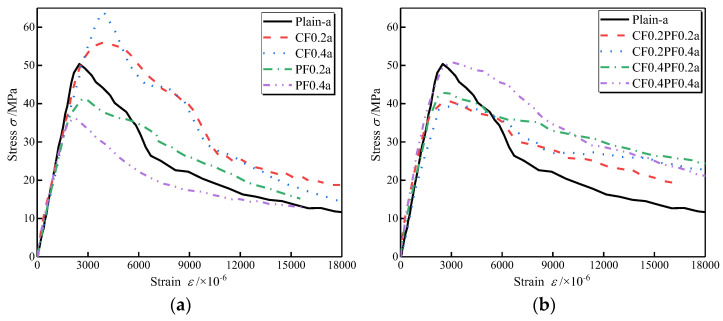
Effects of CF and PF on the compressive stress–strain curves of LWAC (W/B = 0.27): (**a**) fibers in single form; (**b**) fibers in hybrid form.

**Figure 5:**
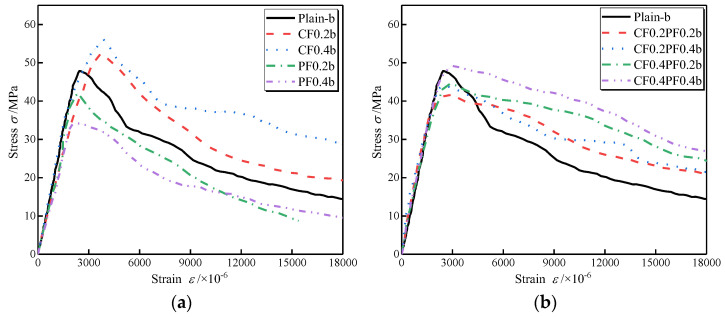
Effects of CF and PF on the compressive stress–strain curves of LWAC (W/B = 0.3): (**a**) fibers in single form; (**b**) fibers in hybrid form.

Figure 4 and Figure 5 present the effects of different fiber combinations on the compressive stress–strain behavior of LWAC with W/B of 0.27 and 0.3, respectively. All experimental stress–strain curves show a similar shape in ascending and descending branches, but the critical points referred to in Figure 3 change with changes in fiber dosages and W/B. As shown in the figure, the initial linear stage of the stress–strain curve shows no noticeable difference with the addition of fibers. However, the nonlinear stage of the ascending branch is significantly affected. When adding fibers to the LWAC, the drop in the ascending branch is flatter, and the nonlinear stage of the ascending branch is wider than that of the plain LWAC, indicating a larger energy dissipation capacity for the FLWAC. For LWAC containing a single type of fiber, the gradient of the descending branch is close to that of the corresponding plain LWAC, as plotted in Figure 4a and Figure 5a. Instead, as presented in Figure 4b and Figure 5b, the descending parts in HFLWAC become much flatter than those of plain LWAC, suggesting an increase in energy dissipation capacity when referring to the post-peak portion.

### 3.3. Stress–Strain Characteristics

Table 5 contains the characteristic values of the experimental stress–strain curves, including peak stress, critical strain, initial tangential elastic modulus, and peak secant elastic modulus. The cubic compressive strength (*f*_cu_) obtained from the tests is also listed in Table 5. As can be seen, both axial and cubic compressive strengths decreased with fiber addition, except with the addition of only carbon fiber. The probable reason for this may be the high elastic modulus and tensile strength of the carbon fiber. Thus, a load-bearing skeleton can be formed in concrete. However, the low elastic modulus of polypropylene fiber caused more defects in the compactness of concrete. As can also be seen, with the decrease in W/B, the compressive strength of concrete increases, which can be attributed to the increase in compactness of the hydrated cement paste [25]. As expected, when W/B decreases from 0.30 to 0.27, the axial compressive strength of plain concrete increases from 47.87 MPa to 50.38 MPa. Meanwhile, the compressive strength of FLWAC in Series 1 is higher than that of corresponding concrete in Series 2.

The critical strain is defined as the strain at the peak point, from which the vertical deformability of the specimen can be evaluated. The critical strain of FLWAC was in the range of 0.00231~0.00394, with an average of 0.002939, which is slightly higher than that of the plain LWAC. Besides that, it can be concluded that the critical strain of LWAC is higher than the critical strain of 0.002 for NWC, as reported in the Chinese Specification GB 50010-2010 [26].

The elastic modulus is extensively used to evaluate the deformability and stiffness of concrete [27]. As provided in Table 5, the *E*_c_ and *E*_0_ for plain LWAC and FLWAC range from 18.6 GPa to 23.4 GPa and 14.2 GPa to 20.2 GPa, respectively, lower than the *E*_c_ of NWC at the comparable strength specified by CEB-FIP Model Code [28]. This phenomenon might be correlated with the porous structure of the LWA, thereby increasing the deformability of aggregate, which is directly responsible for the reduction in the elastic modulus [29]. Moreover, it is believed that the addition of fibers to LWAC reduces its elastic modulus as a result of the interference of fibers with the compactness of concrete. The ratio of *E*_c_ to *E*_0_ can reflect the curvature of the ascending branch in the stress–strain curve. In other words, the value of *E*_c_/*E*_0_ demonstrates the characteristic of nonlinearity in the ascending branch. The larger the *E*_c_/*E*_0_, the more significant the nonlinearity. As can be observed, the plain LWAC has an *E*_c_/*E*_0_ of 1.157 and 1.186, indicating the apparent linearity of LWAC. Besides that, *E*_c_/*E*_0_ increases with the addition of fibers, suggesting significant nonlinearity in the ascending branch of the FLWAC.

### 3.4. Toughness

**Figure 6:**
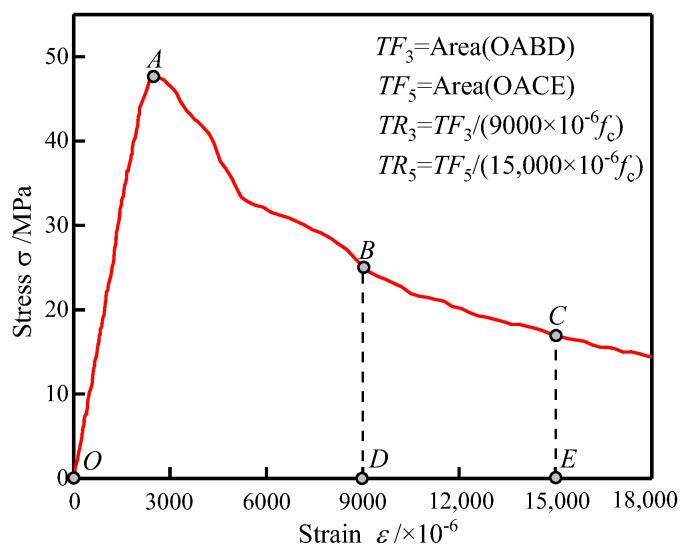
Definitions of toughness and specific toughness.

The toughness is calculated as the area under the stress–strain curve up to the specified strain, representing the concrete’s energy absorption capacity and ductility. The specified strain was set to 0.009 and 0.015, which is 3 and 5 times the ultimate strain following the ACI 318 standard [30], which is sufficient for evaluating the post-peak deformation following Fanella and Naaman [31]. The specific toughness is defined as the toughness ratio to peak strength since the toughness is affected by the compressive strength. Therefore, the specific toughness is considered a better measure to reflect the effect of fibers on the energy absorption capacity. The definitions of toughness and specific toughness are shown in Figure 6. The toughness and specific toughness are calculated using Equations (3) and (4), respectively:

(3) $$TF_{i}=\int_{0}^{\varepsilon_{i}}\sigma \left(\varepsilon \right)d\varepsilon$$

(4) $$TR_{i}=\frac{\int_{0}^{\varepsilon_{i}}\sigma \left(\varepsilon \right)d\varepsilon }{f_{c}\varepsilon_{i}}=\frac{TF_{i}}{f_{c}\varepsilon_{i}}$$

where *ε*_i_ represents *ε*_1_ and *ε*_2_, corresponding to a specific strain of 0.009 and 0.015; *TF*_1_ and *TF*_2_ are the toughness corresponding to the specific strain of *ε*_1_ and *ε*_2_; *TR*_1_ and *TR*_2_ are the specific toughness corresponding to the specific strain *ε*_1_ and *ε*_2_.

Table 6 shows the compressive toughness and specific toughness of LWAC, with which the effects of the fiber combination and W/B on the energy absorption capacity of LWAC can be exhibited. In the case of LWAC in Series 1, an initial increase followed by a decrease in toughness and specific toughness can be observed following the increase in polypropylene fiber content. The lower compressive strength of PF0.2a and PF0.4a may be the main reason for the decrease in toughness. The addition of carbon fiber is conducive to increasing the energy absorption capacity and the ductility of LWAC. In the case of LWAC in Series 2, an increase in both toughness and specific toughness can be observed with the increase in carbon fiber content. The toughness and specific toughness of PF0.2b and PF0.4b approach those of plain LWAC. It can be inferred that carbon fiber has a superior effect on the post-peak behavior of LWAC as compared to polypropylene fiber. Moreover, by comparing Plain 1 with Plain 2, the toughness and specific toughness for Series 2 are more significant than the corresponding specimens in Series 1. Therefore, the reduction in W/B (from 0.3 to 0.27) reduces the effect of fiber addition on the toughness of concrete.

When LWAC contained carbon–polypropylene hybrid fibers, an increase in toughness and specific toughness was observed. The percentage of the specific toughness increase is shown in Table 6 with the corresponding plain concrete as control concrete. For instance, HFLWAC shows an increase in *TR*_5_ of 42.6~56.3% and 26.2~38.3% for W/B of 0.27 and 0.30, respectively. Besides that, CF0.4PF0.2 and CF0.4PF0.4 show a specific toughness larger than CF0.2, which shows that the carbon fiber has a better effect on the energy absorption capacity than polypropylene fiber does, similar to the results in single-fiber-reinforced LWAC.

### 3.5. Assessment of Synergy

With an appropriate mixing range, two or more different fibers in the concrete can derive benefits from each of the individual fibers and exhibit a positive synergistic effect, enhancing the material properties of concrete so that they are far superior to a single fiber [32,33]. To assess the effect of hybridization of carbon and polypropylene fibers on toughness, the synergistic effect coefficients were evaluated following the method suggested by Wang et al. [18] as follows:

(5) $$\alpha_{x-1}=\frac{\beta_{\mathrm{CF}-\mathrm{PF}}+\beta_{\min(\mathrm{CF},\mathrm{PF})}}{\beta_{\min(\mathrm{CF},\mathrm{PF})}+\beta_{\max(\mathrm{CF},\mathrm{PF})}}$$

(6) $$\alpha_{x-2}=\frac{\beta_{\mathrm{CF}-\mathrm{PF}}+\beta_{\max(\mathrm{CF},\mathrm{PF})}}{\beta_{\min(\mathrm{CF},\mathrm{PF})}+\beta_{\max(\mathrm{CF},\mathrm{PF})}}$$

where *α*_*x*−1_ and *α*_*x*−2_ are introduced to represent the synergistic effect coefficients, and x is one of the material properties that need to be identified. In this study, the effect of synergy on toughness was predicted; *α*_t3−1_ and *α*_t3−2_ represent the synergistic effect coefficients of *TR*_3_, and *α*_t5−1_ and *α*_t5−2_ are the synergistic effect coefficients of *TR*_5_. The parameter *β* is the toughness enhancement ratio of FLWAC to the corresponding plain LWAC, *β*_CF−PF_ is the toughness enhancement ratio of HFLWAC, and *β*_min(CF,PF)_ and *β*_max(CF,PF)_ are the minimum and maximum enhancement ratios of individual carbon and polypropylene fibers that compose the hybrid fibers, respectively. The idea behind this method is that when *α*_*x*−1_ > 1, the combination of fibers produces a positive synergy; meanwhile, when *α*_*x*−1_ < 1, but *α*_*x*−2_ > 1, it is a positive synergistic effect, and when *α*_*x*−2_ < 1, it is a negative synergistic effect.

The synergistic effect coefficients noted for toughness are presented in Table 7. As indicated in the table, all specimens exhibited positive synergistic effects, which represents that the hybridization of carbon and polypropylene fibers leads to toughness enhancement that is numerically more significant than the sum of the individual fibers. Polypropylene fiber did not add much to the toughness but shows effectiveness in contributing to the toughness when combined with carbon fiber. In addition, hybridization was less effective at higher fiber dosages in both Series 1 and Series 2. For instance, in Series 1, the best performance was obtained with carbon fiber at 0.2% and polypropylene fiber at 0.4%. Besides that, it can be seen from Table 7 that W/B shows little influence on the reinforcement of fiber in LWAC.

## 4. Compressive Stress–Strain Model

### 4.1. Modeling of Compressive Strength

The relationship between *f*_cu_ and *f*_c_ was basically linear, and a formula is proposed with relevant test data, as follows:

(7) $$f_{c}=0.82f_{\mathrm{cu}}+2.01$$

**Figure 7:**
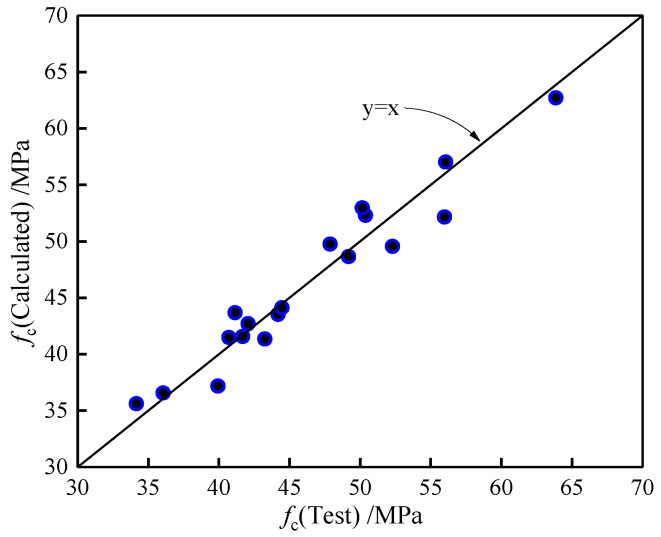
Comparison of *f*_c_ between experimental and calculated values.

A comparison of the calculated results with the test values is shown in Figure 7. As can be observed, the calculated values match the experimental values well. Then, Equation (7) can be used to calculate the *f*_c_ of LWAC containing carbon and polypropylene fibers.

### 4.2. Modeling of Critical Strain at Peak Compressive Stress

In order to analyze the influence of the two types of fibers on the critical strain, the critical strain ratio (*ε*_c_/*ε*_0_) is taken to compensate for the effect of W/B, where *ε*_c_/*ε*_0_ is the ratio of critical strain for FLWAC (*ε*_c_) to the critical strain for the corresponding plain LWAC (*ε*_0_). As Section 3.2 stated, the FLWAC with the same mix design as the corresponding plain LWAC contains different fiber combinations.

It can be observed in Table 5 that *ε*_c_/*ε*_0_ increases with the increase in *V*_CF_ but slightly decreases with the increase in *V*_PF._ An analytical equation for describing the relationship between the critical strain ratio and fiber volume fraction was obtained by fitting experimental data, as shown in Equation (8) (*R*^2^ = 0.741):

(8) $$\varepsilon_{c}/\varepsilon_{0}=1+0.1281{\left(RI\right)}_{\mathrm{CF}}-0.0613{\left(RI\right)}_{\mathrm{PF}}-27150{\left(RI\right)}_{\mathrm{CF}}{\left(RI\right)}_{\mathrm{PF}}$$

where *ε*_c_ is the critical strain of FLWAC, and *ε*_0_ is the critical strain of the corresponding plain LWAC; for example, in Series 1, *ε*_0_ is the critical strain of specimen Plain-a, and (*RI*)_CF_ and (*RI*)_PF_ are the reinforcing indexes (calculated as the aspect ratio multiplied by the volume fraction) of carbon fiber and polypropylene fiber, respectively. The critical strain of plain LWAC can be modeled using the following analytical expression proposed by Lim and Ozbakkaloglu [17]:

(9) $$\varepsilon_{0}=\frac{f_{c}^{0.225k_{d}}}{1000}{\left(\frac{152}{D}\right)}^{0.1}{\left(\frac{2D}{H}\right)}^{0.13}$$

where *k*_d_ is the parameter to allow for density; *D* is the diameter of the specimen (*D* = 100 mm); and *H* is the height of the specimen (*H* = 300 mm). When *ε*_0_ is known, *ε*_c_ for FLWAC can be calculated. 

**Figure 8:**
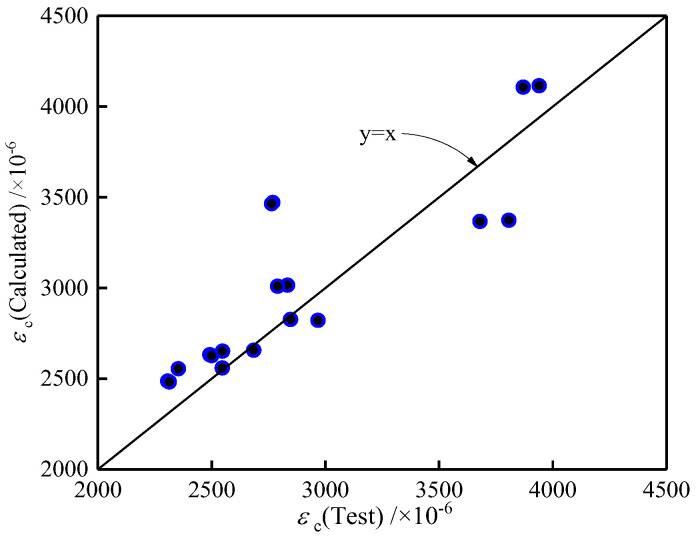
Comparison of *ε*_c_ between experimental and calculated values.

The values of *ε*_c_ calculated by Equations (8) and (9) based on (*RI*)_CF_ and (*RI*)_PF_ in this study are compared with the test values of *ε*_c_ in Figure 8. As can be seen from the comparison, the calculated values are in good agreement with the test results. Then, Equations (8) and (9) can be used to model the critical strain of LWAC containing single and hybrid carbon and polypropylene fibers.

### 4.3. Modeling of the Stress–Strain Curve

#### 4.3.1. Existing Models

As presented in Table 8, five typical existing stress–strain models, including those developed by Carreira and Chu [12], Abbass et al. [34], Ou et al. [35], Oliveira Júnior et al. [36], and Wang et al. [18], were applied to predict the compressive behavior of FLWAC.

After reviewing these models, it is recognized that the five existing models, except for the model of Wang et al. [18], were modified based on the Carreira and Chu model that was initially used to describe the behavior of plain concrete in compression. Abbass et al. [34] and Ou et al. [35] modified the parameter *β* with the fiber reinforcing index, with which the fiber characteristic can be considered in the models. In the model established by Oliveira Júnior et al. [36], the material parameter *β* contains the fiber volume fraction and compressive strength. Wang et al.’s model was derived for the axial compressive behavior of basalt–polypropylene hybrid fiber reinforced concrete. Since the reinforcing index of carbon fiber in this study is similar to that of basalt fibers in [17], this model was applied to the experimental data of LWAC containing carbon and polypropylene fibers.

**Figure 9:**
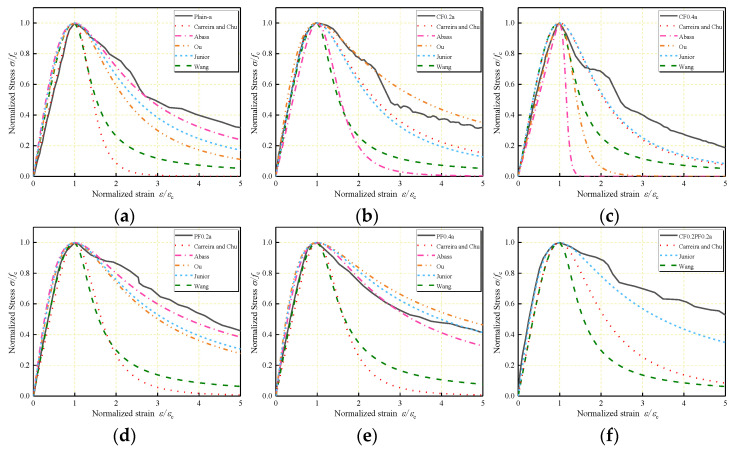

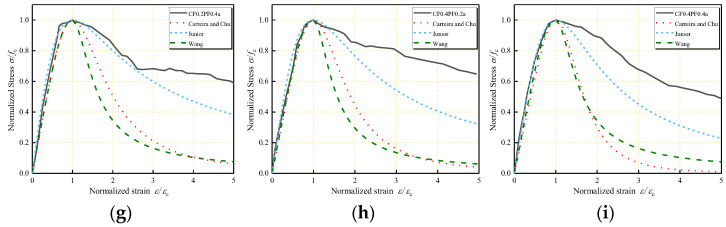
Application of existing stress–strain models to the data of: (**a**) Plain–a; (**b**) CF0.2a; (**c**) CF0.4a; (**d**) PF0.2a; (**e**) PF0.4a; (**f**) CF0.2PF0.2a; (**g**) CF0.2PF0.4a; (**h**) CF0.4PF0.2a; (**i**) CF0.4PF0.4a.

**Figure 10:**
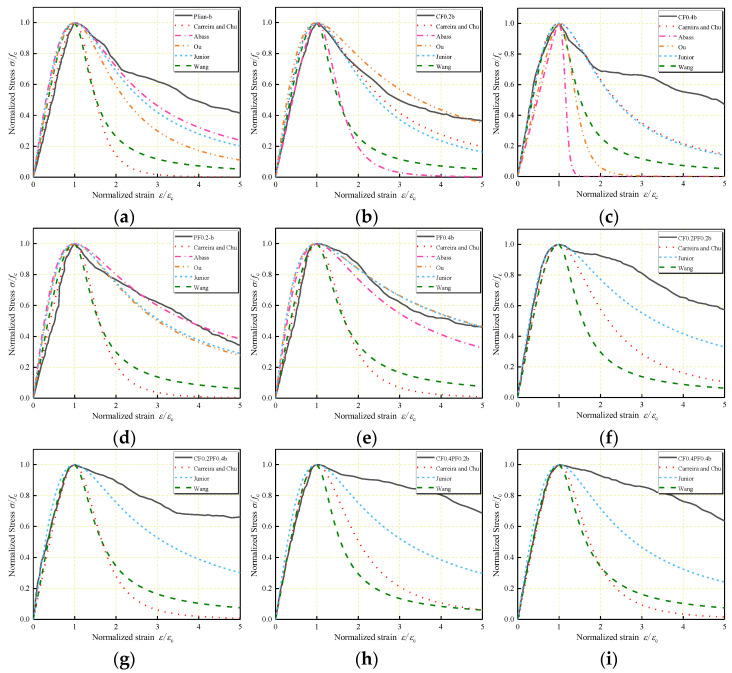
Application of existing stress–strain models to the data of: (**a**) Plain–b; (**b**) CF0.2b; (**c**) CF0.4b; (**d**) PF0.2b; (**e**) PF0.4b; (**f**) CF0.2PF0.2b; (**g**) CF0.2PF0.4b; (**h**) CF0.4PF0.2b; (**i**) CF0.4PF0.4b.

Comparisons of the predicted stress–strain curves with the experimental curves for Series 1 and Series 2 are shown in Figure 9 and Figure 10. As can be observed, the ascending branch in stress–strain curves predicted by the existing models is in close agreement with the experimental results. In the descending branch, Carreira and Chu’s model and Wang et al.’s model underestimate the post-peak region of the curves. Abbass et al.’s model can be used to effectively describe the behavior of PFLWAC but underestimates the behavior of CFLWAC. However, Abbass et al.’s model and Ou et al.’s model can only be used in single-fiber-reinforced concrete for just one variable considered in the parameter *β*. In Oliveira Junior et al.’s model, to express the stress–strain curves of HFLWAC, the volume fraction of fiber used in the parameter *β* was calculated by the sum of the two types of fibers. The curves for single-fiber-reinforced LWAC that Oliveira Júnior et al. [36] predicted were close to the experimental curves; however, the curves for HFLWAC were underestimated.

#### 4.3.2. New Proposal for the Stress–Strain Curve of FLWAC

As previously mentioned, Carreira and Chu’s model (1985) gives a relatively good representation of the ascending portion. Therefore, the stress–strain model generated by Carreira and Chu (1985) and later improved by Wee et al. (1986) was chosen to predict the stress–strain curve of LWAC. The parameters of this model were further modified to predict the stress–strain behavior of carbon- and/or polypropylene-fiber-reinforced LWAC. The model is represented by the following:

(10) $$\sigma =f_{c}\left(\frac{k_{1}\beta \left(\varepsilon /\varepsilon_{c}\right)}{k_{1}\beta -1+{\left(\varepsilon /\varepsilon_{c}\right)}^{k_{2}\beta }}\right)$$

(11) $$\beta =\frac{1}{1-\left(f_{c}/\varepsilon_{c}E_{c}\right)}$$

where *β* is the material parameter, which can be obtained from Equation (11); the parameter *k*_1_ is applied to the numerator, and in the first term of the denominator, *k*_2_ is applied to the exponent of the last term of the denominator.

According to the above analysis, the regression equations of parameters *k*_1_ and *k*_2_ are determined by using (*RI*)_CF_, (*RI*)_PF_, and *β* as variables, as follows:

(12) $$k_{1}=1.2524-0.0197{\left(RI\right)}_{\mathrm{CF}}-0.3224{\left(RI\right)}_{\mathrm{PF}}-0.0875\beta$$

(13) $$k_{2}=1.1476-0.0158{\left(RI\right)}_{\mathrm{CF}}-0.2512{\left(RI\right)}_{\mathrm{PF}}-0.1059\beta$$

Here, *k*_1_ and *k*_2_ can be calculated by Equations (12) and (13), and then the fitted stress–strain curve can be determined by Equation (10). Table 9 shows the experimental and calculated parameters. The COVs are 0.587 and 0.808 for *k*_1_ and *k*_2_; the average ratio is 1.00 and 1.02 for *k*_1_ and *k*_2_, respectively.

### 4.4. Comparison between Experimental Results and the Proposed Model

**Figure 11:**
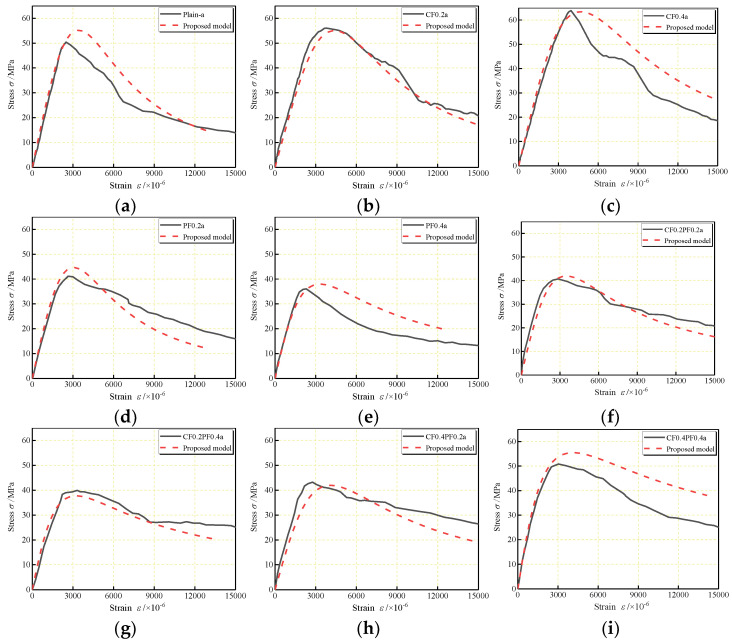
Comparison of calculated complete stress–strain curves for: (**a**) Plain–a; (**b**) CF0.2a; (**c**) CF0.4a; (**d**) PF0.2a; (**e**) PF0.4a; (**f**) CF0.2PF0.2a; (**g**) CF0.2PF0.4a; (**h**) CF0.4PF0.2a; (**i**) CF0.4PF0.4a.

**Figure 12:**
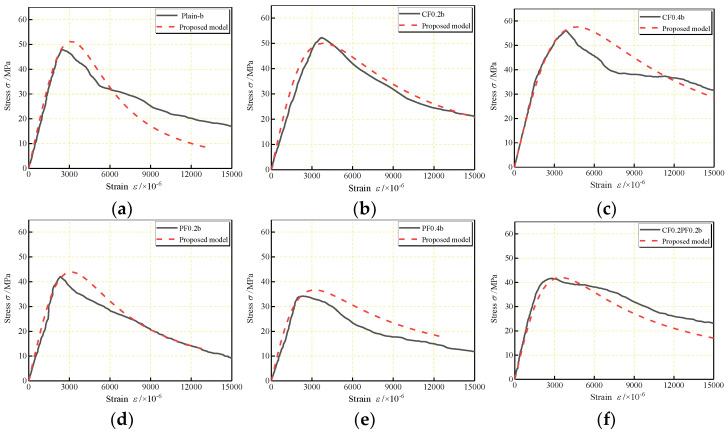

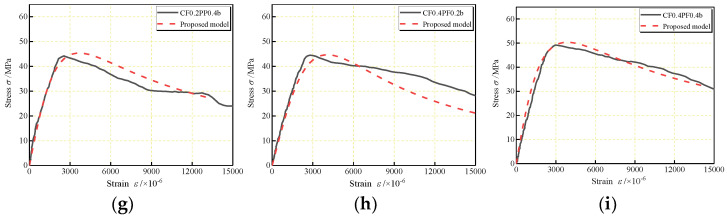
Comparison of calculated complete stress–strain curves for: (**a**) Plain–b; (**b**) CF0.2b; (**c**) CF0.4b; (**d**) PF0.2b; (**e**) PF0.4b; (**f**) CF0.2PF0.2b; (**g**) CF0.2PF0.4b; (**h**) CF0.4PF0.2b; (**i**) CF0.4PF0.4b.

Comparisons of the experimental and analytical stress–strain curves of FLWAC are shown in Figure 11 and Figure 12 for W/B of 0.27 and 0.30, respectively. There is a reasonable adjustment between the analytical ascending branch of the predicted curve and the experimental curve. When referring to the descending branch, a slight difference can be found between experimental results and predicted curves, which can be attributed to the inconsistent manner in which cracks occurred. In both cases, the proposed model shows better accuracy relative to the experimental curve than the existing models.

## 5. Conclusions

The compressive stress–strain relationship of LWAC containing different combinations of carbon and polypropylene fibers and different W/B was investigated experimentally through the compressive testing of prisms. Furthermore, regression formulas of compressive strength and critical strain were derived, and a stress–strain model for HFLWAC was established. Therefore, the following conclusions can be drawn:

The failure modes were less affected by W/B but displayed significant modifications with the addition of fibers. Although CFLWAC and plain LWAC showed similar failure modes with localized concrete crushing, LWAC containing polypropylene fiber maintained integrity during the testing, and no apparent crushing was observed after the test.

Hybrid carbon–polypropylene fibers have better reinforcing effects on LWAC than single fibers. All specimens show positive synergistic effects, demonstrating the positive hybridization effect of carbon and polypropylene fibers.

The axial compressive strength of FLWAC is proportional to *f*_cu_, and a conversion relationship between them was established by the regression of experimental data through Equation (7). An empirical formula of the critical strain of FLWAC based on fiber reinforcing indexes ((*RI*)_CF_ and (*RI*)_PF_) and the critical strain of plain LWAC was proposed through Equations (8) and (9).

All stress–strain curves show a similar trend in their mathematical characteristics. One stress–strain model can be estimated by using the appropriate curve parameter. The relationship between the parameters (*k*_1_ and *k*_2_) and fiber reinforcing indexes ((*RI*)_CF_ and (*RI*)_PF_) is estimated through Equations (10)–(13).

## Figures and Tables

**Table 1:** Properties of LWA.

Bulk Density (kg/m^3^)	Apparent Density (kg/m^3^)		Crushing Strength (MPa)		1 h/24 h Water Absorption (%)			Total Porosity (%)		Particle Size Distribution (%)				
										2.36~5 mm		5~10 mm	10~16 mm	
860	1512		6.9		2.2/2.6			43.12		11		68	21	
**Chemical Composition**														
SiO_2_ (%)	Al_2_O_3_ (%)	Fe_2_O_3_ (%)		TiO_2_ (%)		CaO (%)	MgO (%)		SiO_3_ (%)		Alkalis as Na_2_O (%)			LOI (%)
65.4	15.9	4.2		0.7		2.4	3.7		0.23		3.8			3.67

**Table 2:** Properties of fibers.

Fiber Details	Carbon Fiber	Polypropylene Fiber
View	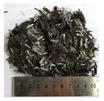	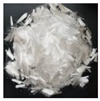
Fiber shape	Straight, filaments	Straight, fibrillated
Cut length (*L*_f_) (mm)	8~10	15~22
Diameter (*D*_f_) (μm)	7	80
Aspect ratio (*L*_f_/*D*_f_)	1100	225
Specific gravity (g/cm^3^)	1.8	0.91
Elongation (%)	2.1	17
Tensile strength (MPa)	4000	>400
Elastic modulus (GPa)	240	22
Water absorption	<1% by weight	Nil

**Table 3:** The mix proportions of plain LWAC (kg/m^3^).

Mixture	W/B	Cement	Silica Fume	Fly Ash	LWA	Sand	Water	Superplasticizer
Plain–a	0.27	440	44	66	603	684	148.5	6.8
Plain–b	0.3	440	44	66	578	667	165	5.2

**Table 4:** Fiber addition in LWAC mixtures.

Fiber	W/B	Carbon Fiber/(%)		Polypropylene Fiber/(%)		Carbon–Polypropylene Hybrid Fibers/(%)			
		0.2	0.4	0.2	0.4	0.2/0.2	0.2/0.4	0.4/0.2	0.4/0.4
Mix code	0.27	CF0.2a	CF0.4a	PF0.2a	PF0.4a	CF0.2PF0.2a	CF0.2PF0.4a	CF0.4PF0.2a	CF0.4PF0.4a
	0.3	CF0.2b	CF0.4b	PF0.2b	PF0.4b	CF0.2PF0.2b	CF0.2PF0.4b	CF0.4PF0.2b	CF0.4PF0.4b

**Table 5:** Characteristic values of compressive stress–strain curves of LWAC.

Specimen	W/B	*V*_CF_ (%)	*V*_PF_ (%)	*f*_cu_ (MPa)	*f*_c_ (MPa)	*ε*_c_ (10^−6^)	*ε*_c_/*ε*_0_	*E*_c_ (GPa)	*E*_0_ (GPa)	*E*_c_/*E*_0_
Plain–a	0.27	0	0	61.34 (0.041)	50.38	2492	1.000	23.4	20.2	1.157
CF0.2a		0.2	0	61.17 (0.038)	55.97	3807	1.528	22.9	14.7	1.558
CF0.4a		0.4	0	74.03 (0.054)	63.86	3940	1.581	23.1	16.2	1.425
PF0.2a		0	0.2	50.83 (0.045)	41.15	2547	1.022	20.1	16.2	1.244
PF0.4a		0	0.4	42.15 (0.036)	36.05	2307	0.926	19.4	15.6	1.241
CF0.2PF0.2a		0.2	0.2	48.15 (0.062)	40.71	2834	1.137	20.7	14.4	1.441
CF0.2PF0.4a		0.2	0.4	42.90 (0.049)	39.93	2685	1.077	20.8	14.9	1.399
CF0.4PF0.2a		0.4	0.2	47.98 (0.039)	43.25	2769	1.111	21.1	15.6	1.351
CF0.4PF0.4a		0.4	0.4	62.15 (0.061)	50.15	2847	1.142	22.2	17.6	1.260
Plain–b	0.3	0	0	58.24 (0.030)	47.87	2500	1.000	22.7	19.1	1.186
CF0.2b		0.2	0	57.99 (0.033)	52.29	3680	1.472	23.2	14.2	1.633
CF0.4b		0.4	0	67.11 (0.031)	56.06	3870	1.548	22.4	14.5	1.546
PF0.2b		0	0.2	49.64 (0.041)	42.07	2354	0.942	21.8	17.9	1.220
PF0.4b		0	0.4	40.99 (0.064)	34.16	2314	0.926	18.6	14.8	1.260
CF0.2PF0.2b		0.2	0.2	48.26 (0.019)	41.68	2790	1.116	22.0	14.9	1.473
CF0.2PF0.4b		0.2	0.4	50.65 (0.030)	44.19	2548	1.019	21.7	17.3	1.251
CF0.4PF0.2b		0.4	0.2	51.36 (0.047)	44.46	2764	1.106	22.5	16.1	1.399
CF0.4PF0.4b		0.4	0.4	56.88 (0.028)	49.18	2968	1.187	21.3	16.6	1.285

**Table 6:** Toughness and specific toughness of LWAC.

Specimen	*σ*_0.009_ (MPa)	*σ*_0.015_ (MPa)	Toughness		Specific Toughness (%)	
			*TF*_3_	*TF*_5_	*TR*_3_	*TR*_5_
Plain-a	22.09	13.88	0.2933	0.3954	0.6468 (-)	0.5232 (-)
CF0.2a	39.58	20.73	0.3882	0.5465	0.7703	0.6506
CF0.4a	37.83	18.58	0.3888	0.5419	0.6764	0.5657
PF0.2a	26.01	15.98	0.2801	0.4030	0.7563	0.6529
PF0.4a	17.23	13.19	0.2177	0.3082	0.6701	0.5699
CF0.2PF0.2a	29.32	21.71	0.3070	0.4591	0.8379 (29.5%)	0.7518 (43.7%)
CF0.2PF0.4a	28.45	26.08	0.3018	0.4650	0.8399 (29.9%)	0.7764 (48.4%)
CF0.4PF0.2a	34.57	27.78	0.3206	0.5049	0.8656 (33.8%)	0.8178 (56.3%)
CF0.4PF0.4a	34.63	25.69	0.3664	0.5413	0.8007 (23.8%)	0.7097 (35.6%)
Plain-b	25.15	16.97	0.2919	0.4145	0.6775 (-)	0.5772 (-)
CF0.2b	31.79	21.14	0.3317	0.4822	0.7049	0.6148
CF0.4b	38.02	31.60	0.3651	0.5600	0.7236	0.6897
PF0.2b	20.28	9.18	0.2525	0.3371	0.6669	0.5342
PF0.4b	17.73	11.77	0.2159	0.3046	0.7022	0.5944
CF0.2PF0.2b	31.94	23.03	0.3116	0.4712	0.8322 (22.8%)	0.7551 (30.8%)
CF0.2PF0.4b	30.25	23.96	0.3128	0.4827	0.7866 (16.1%)	0.7282 (26.2%)
CF0.4PF0.2b	37.65	28.16	0.3280	0.5296	0.8198 (21.0%)	0.7942 (37.6%)
CF0.4PF0.4b	42.12	30.89	0.3642	0.5871	0.8229 (21.5%)	0.7985 (38.3%)

Note: the data in parentheses show the percentage of specific toughness increase over that of plain concrete.

**Table 7:** Specific toughness synergistic coefficient.

Specimen	*α*_t3−1_	*α*_t5−1_
CF0.2PF0.2a	1.053	1.076
CF0.2PF0.4a	1.087	1.103
CF0.4PF0.2a	1.132	1.207
CF0.4PF0.4a	1.092	1.127
CF0.2PF0.2b	1.093	1.122
CF0.2PF0.4b	1.058	1.094
CF0.4PF0.2b	1.069	1.085
CF0.4PF0.4b	1.070	1.084

**Table 8:** Existing stress–strain models for concrete in compression.

Models	Fitting Expressions	Crucial Parameters
Carreira and Chu, 1985	$\sigma =f_{c}\left(\frac{\beta \left(\varepsilon /\varepsilon_{c}\right)}{\beta -1+{\left(\varepsilon /\varepsilon_{c}\right)}^{\beta }}\right)$	$\beta =1/\left(1-\left(f_{c}/\varepsilon_{c}E_{c}\right)\right)$
Abbass et al., 2018	$\sigma =f_{c}\left(\frac{\beta \left(\varepsilon /\varepsilon_{c}\right)}{\beta -1+{\left(\varepsilon /\varepsilon_{c}\right)}^{\beta }}\right)$	$\beta =1.401{\left(RI\right)}^{2}-1.56(RI)+2.42$
Ou et al., 2012	$\sigma =f_{c}\left(\frac{\beta \left(\varepsilon /\varepsilon_{c}\right)}{\beta -1+{\left(\varepsilon /\varepsilon_{c}\right)}^{\beta }}\right)$	$\beta =0.71{\left(RI\right)}^{2}-2(RI)+3.05$
Júnior et al., 2010	$\sigma =f_{c}\left(\frac{\beta \left(\varepsilon /\varepsilon_{c}\right)}{\beta -1+{\left(\varepsilon /\varepsilon_{c}\right)}^{\beta }}\right)$	$\beta =\left(0.0536-0.5754V_{f}\right)f_{c}$
Wang et al., 2019	Ascending branch: $\sigma =f_{c}\left(a_{1}\left(\varepsilon /\varepsilon_{c}\right)+\left(6-5a_{1}\right){\left(\varepsilon /\varepsilon_{c}\right)}^{5}+\left(4a_{1}-5\right){\left(\varepsilon /\varepsilon_{c}\right)}^{6}\right)$ Descending branch: $\sigma =f_{c}\left(\frac{\left(\varepsilon /\varepsilon_{c}\right)}{\alpha {\left(\varepsilon /\varepsilon_{c}-1\right)}^{2}+\varepsilon /\varepsilon_{c}}\right)$	$a_{1}=1.417+0.697V_{\mathrm{BF}}-6.699V_{\mathrm{PPF}}$ $\alpha =5.638+24.01V_{\mathrm{BF}}-468.34V_{\mathrm{PPF}}$

**Table 9:** Comparison of calculated values with the experimental values.

Specimen	*V*_CF_ (%)	*V*_PF_ (%)	*β*	*k*_1_			*k*_2_		
				Experimental	Calculated	Ratio	Experimental	Calculated	Ratio
Plain–a	0	0	7.3508	0.56	0.6092	0.92	0.3833	0.3691	1.04
CF0.2a	0.2	0	2.7933	0.9546	0.9646	0.99	0.8572	0.8169	1.05
CF0.4a	0.4	0	3.3518	1.071	0.8724	1.23	0.8793	0.7230	1.22
PF0.2a	0	0.2	5.0967	0.4895	0.6614	0.74	0.4066	0.4948	0.82
PF0.4a	0	0.4	5.1409	0.4429	0.5124	0.86	0.4139	0.3771	1.10
CF0.2PF0.2a	0.2	0.2	3.2675	0.549	0.7781	0.71	0.5332	0.6537	0.82
CF0.2PF0.4a	0.2	0.4	3.5085	0.5538	0.6119	0.90	0.5083	0.5151	0.99
CF0.4PF0.2a	0.4	0.2	3.8499	0.5331	0.6838	0.78	0.4493	0.5572	0.81
CF0.4PF0.4a	0.4	0.4	4.8419	0.4509	0.4519	1.00	0.397	0.3391	1.17
Plain–b	0	0	6.3908	0.6855	0.6932	0.99	0.4144	0.4708	0.88
CF0.2b	0.2	0	2.5804	1.194	0.9833	1.21	0.9684	0.8395	1.15
CF0.4b	0.4	0	2.8304	0.812	0.9181	0.88	0.7265	0.7782	0.93
PF0.2b	0	0.2	5.5495	0.7507	0.6218	1.21	0.4665	0.4469	1.04
PF0.4b	0	0.4	4.8467	0.8236	0.5382	1.53	0.5093	0.4083	1.25
CF0.2PF0.2b	0.2	0.2	3.1157	0.6874	0.7914	0.87	0.5717	0.6698	0.85
CF0.2PF0.4b	0.2	0.4	4.9805	0.4463	0.4831	0.92	0.3622	0.3592	1.01
CF0.4PF0.2b	0.4	0.2	3.5076	0.7719	0.7137	1.08	0.5258	0.5934	0.89
CF0.4PF0.4b	0.4	0.4	4.5032	0.5982	0.4816	1.24	0.4146	0.3749	1.11

## Data Availability

The data presented in this study are available on request from the corresponding author.

## Abbreviations

*f*_cu_	Cubic compressive strength/MPa
*f*_c_	Compressive strength corresponding to the peak point of stress–strain curve/MPa
*ε*_c_	Critical strain corresponding to the peak point of the stress–strain curve
*ε*_0_	Critical strain for corresponding plain LWAC
*E*_c_	Initial tangential elastic modulus/GPa
*E*_0_	Peak secant elastic modulus/GPa
*TF*_i_	Toughness
*TR*_i_	Specific toughness
*α*_*x*−i_	Synergistic effect coefficients
